# Supplementary material for: The Landscape of Transmembrane Protein Family Members in Head and Neck Cancers: Their Biological Role and Diagnostic Utility
Source: Cancers (Basel). 2021 Sep 22;13(19):4737. doi: 10.3390/cancers13194737 (PMC8507526; doi:10.3390/cancers13194737)
Supplement: Supplementary file 1 [file cancers-13-04737-s001.zip › cancers-1366944-supplementary.pdf]

cancers

The Landscape of Transmembrane Protein Family Members in Head and Neck Cancers: Their Biological Role and Diagnostic Utility

Oliwia Koteluk, Antonina Bielicka, Żaneta Lemańska, Kacper Jóźwiak, Weronika Klawiter, Andrzej Mackiewicz, Urszula Kazimierczak and Tomasz Kolenda

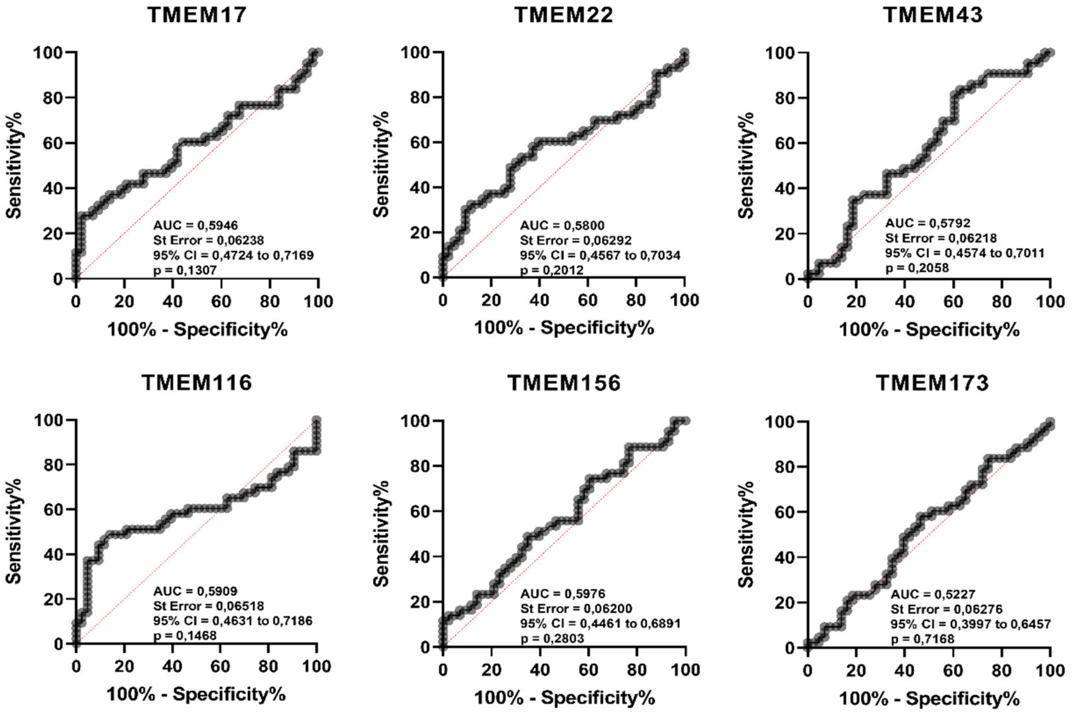

Figure S1. Receiver operating characteristic curve (ROC) analysis of statistically significant *TMEM17*, *TMEM22*, *TMEM43*, *TMEM116*, *TMEM156* and *TMEM173* of HNSCC samples and paired adjacent normal tissues.

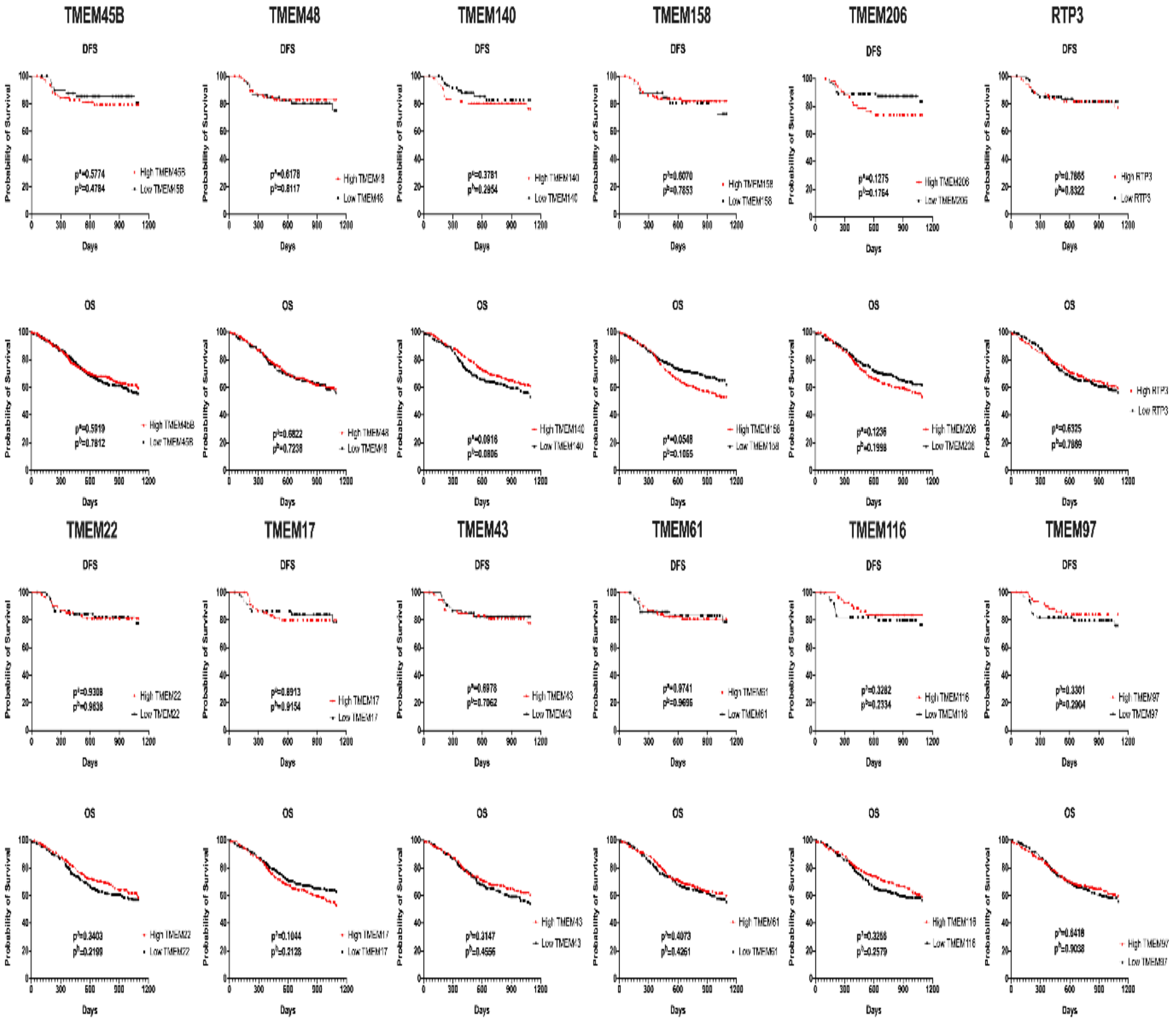

Figure S2. Disease-free survival (DFS) and overall survival (OS) of HNSCC patients (TCGA) depending on *TMEM45B*, *TMEM48*, *TMEM140*, *TMEM158*, *TMEM206*, *RTP3*, *TMEM22*, *TMEM17*, *TMEM43*, *TMEM61*, *TMEM116* and *TMEM97* expression levels; high and low subgroups of patients divided based on mean of expression level; a — Log rank (Mantel-Cox) test, and b — Gehan-Breslow-Wilcoxon test; $p < 0.05$ considered as significant.

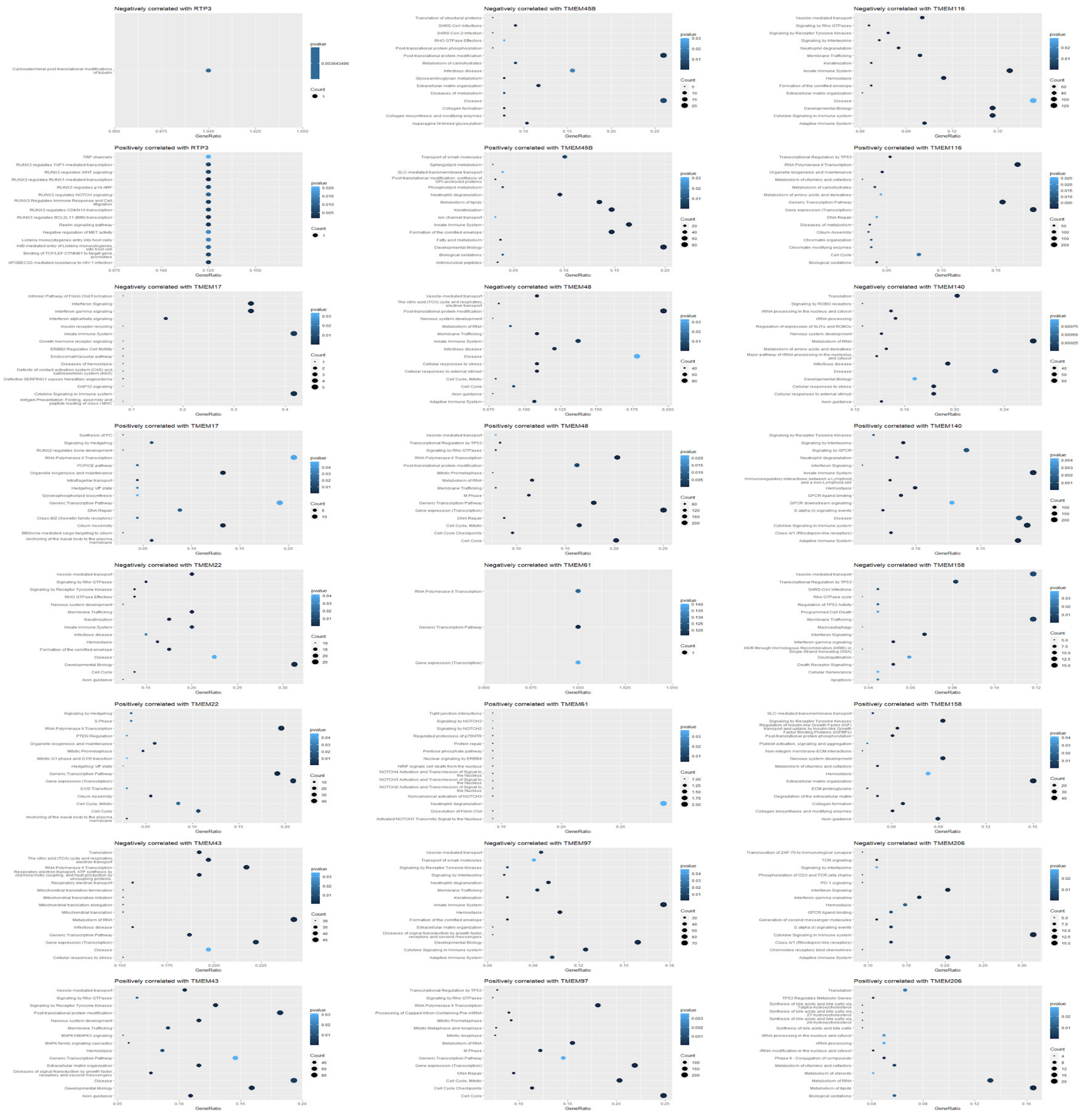

Figure S3. REACTOME pathway enrichment analysis of genes positively ($R > 0.3$) and negatively ($R < -0.3$) correlated with *ANO1*, *RTP3*, *TMEM17*, *TMEM22*, *TMEM43*, *TMEM45B*, *TMEM48*, *TMEM61*, *TMEM97*, *TMEM116*, *TMEM158*, *TMEM206* and *TMEM213*. Fifteen statistically significant ($p < 0.05$) pathways with the highest count are shown.
