# Supplementary material for: The Landscape of Transmembrane Protein Family Members in Head and Neck Cancers: Their Biological Role and Diagnostic Utility
Source: Cancers (Basel). 2021 Sep 22;13(19):4737. doi: 10.3390/cancers13194737 (PMC8507526; doi:10.3390/cancers13194737)
Supplement: Supplementary file 1 [file cancers-13-04737-s001.zip › cancers-1366944-supplementary/cancers-1366944-supplementary.docx]

The Landscape of Transmembrane Protein Family Members in Head and Neck Cancers: Their Biological Role and Diagnostic Utility

Oliwia Koteluk ^1^, Antonina Bielicka, Żaneta Lemańska, Kacper Jóźwiak, Weronika Klawiter, Andrzej Mackiewicz, Urszula Kazimierczak and Tomasz Kolenda


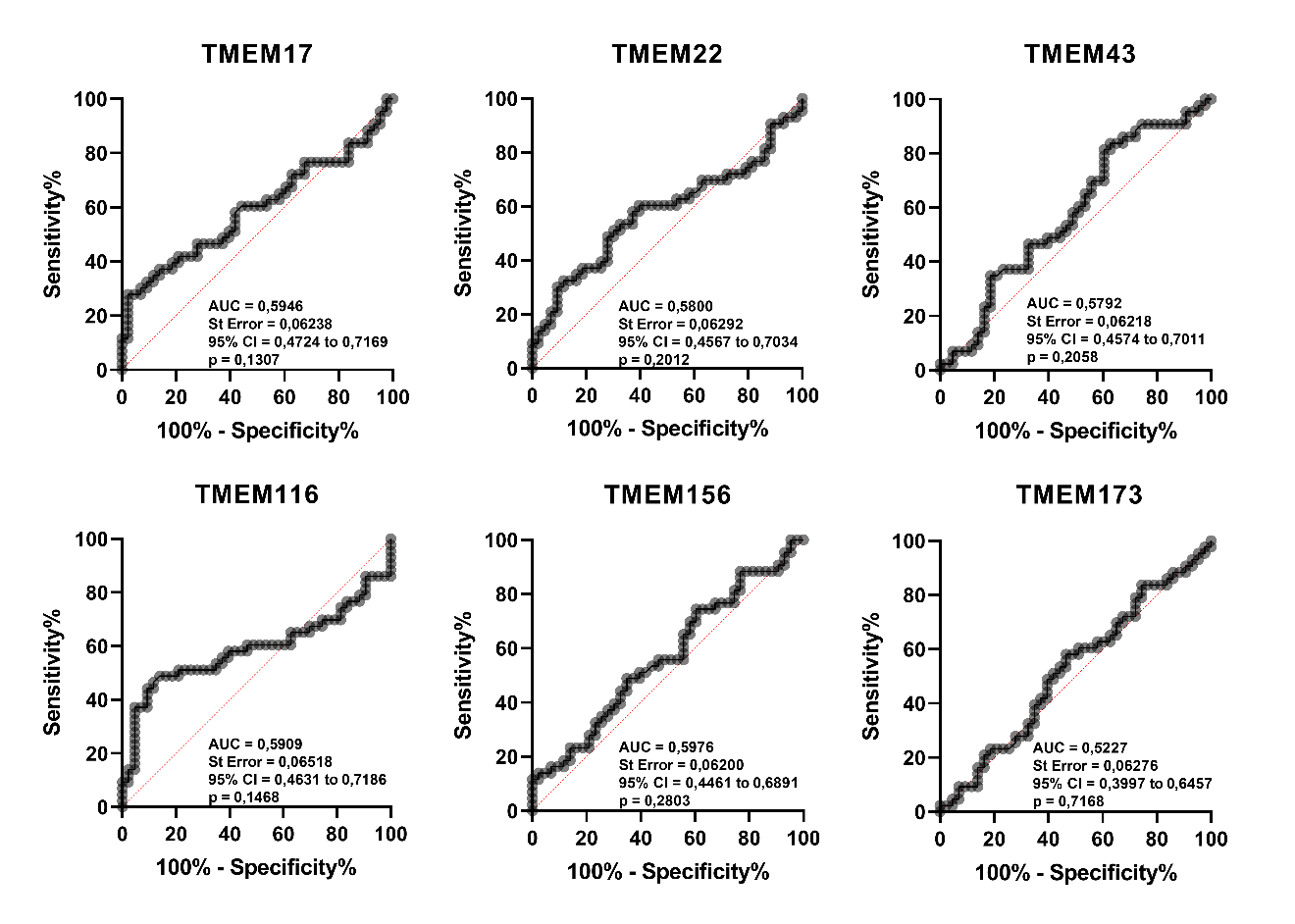


**Figure S1.** Receiver operating characteristic curve (ROC) analysis of statistically significant *TMEM17*, *TMEM22, TMEM43, TMEM116, TMEM156* and *TMEM173* of HNSCC samples and paired adjacent normal tissues.


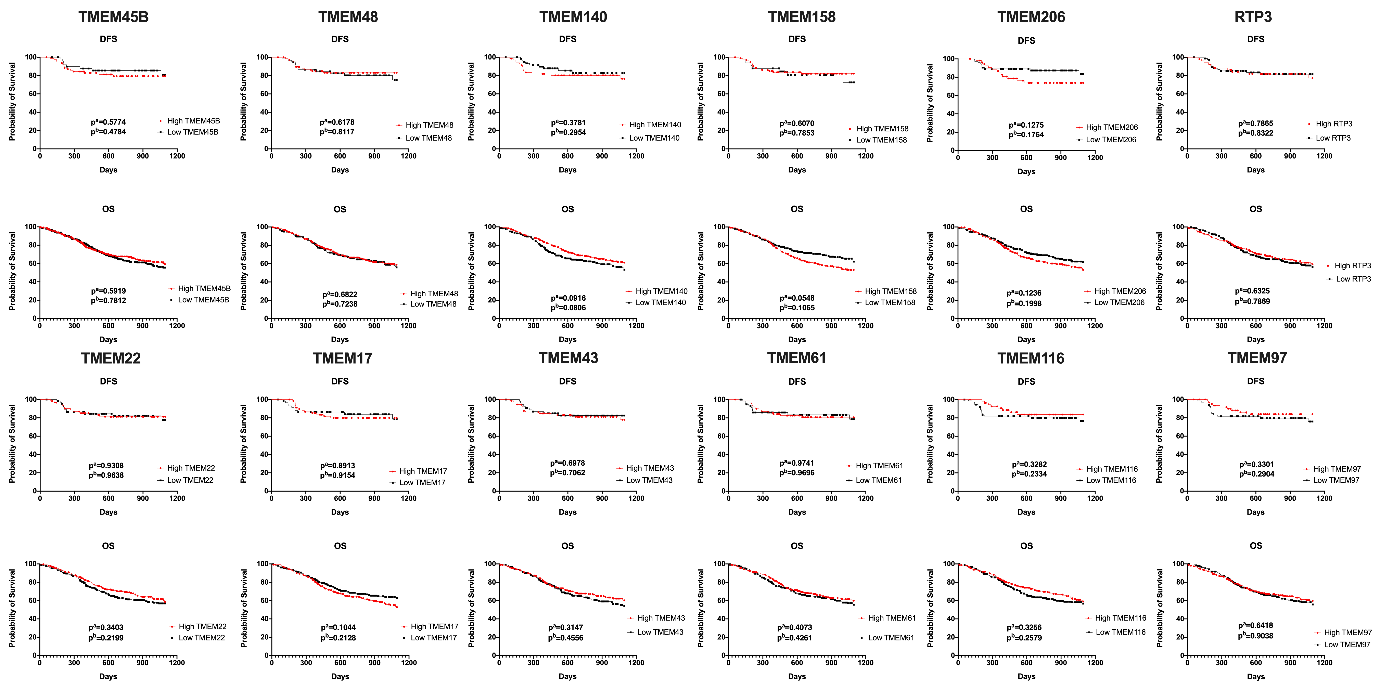


**Figure S2.** Disease-free survival (DFS) and overall survival (OS) of HNSCC patients (TCGA) depending on *TMEM45B, TMEM48, TMEM140, TMEM158, TMEM206, RTP3, TMEM22, TMEM17, TMEM43, TMEM61, TMEM116* and *TMEM97* expression levels; high and low subgroups of patients divided based on mean of expression level; a — Log rank (Mantel-Cox) test, and b — Gehan-Breslow-Wilcoxon test; *p* < 0.05 considered as significant.


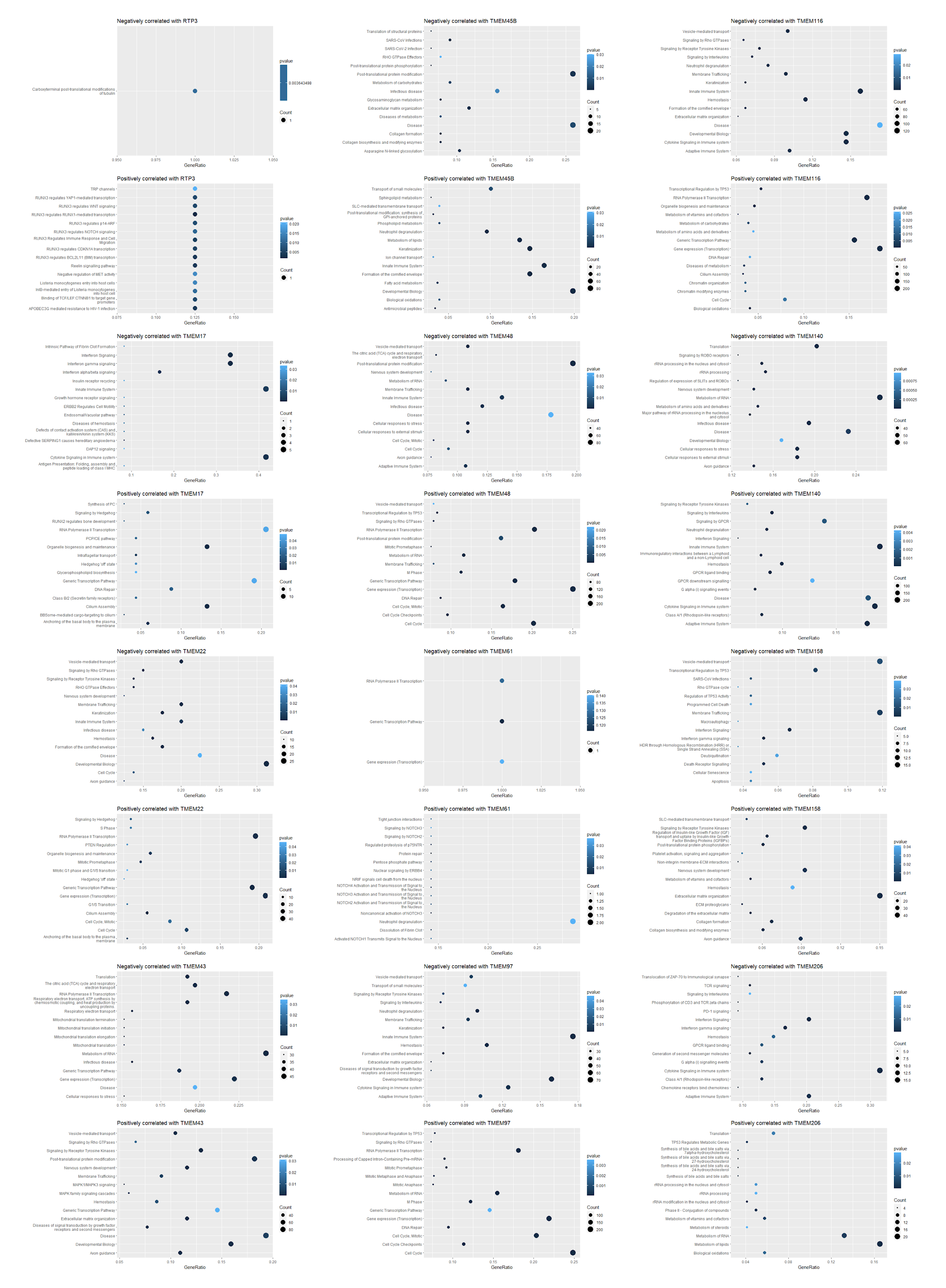


**Figure S3.** REACTOME pathway enrichment analysis of genes positively (R > 0.3) and negatively (R < −0.3) correlated with *ANO1*, *RTP3*, *TMEM17*, *TMEM22*, *TMEM43*, *TMEM45B*, *TMEM48*, *TMEM61*, *TMEM97*, *TMEM116*, *TMEM158*, *TMEM206* and *TMEM213*. Fifteen statistically significant (*p* < 0.05) pathways with the highest count are shown.
